# Thioredoxin TRX*o*1 is involved in ABA perception via PYR1 redox regulation

**DOI:** 10.1016/j.redox.2023.102750

**Published:** 2023-05-26

**Authors:** Sabrina De Brasi-Velasco, Antonio Sánchez-Guerrero, Mari-Cruz Castillo, Didier Vertommen, José León, Francisca Sevilla, Ana Jiménez

**Affiliations:** a Department of Stress Biology and Plant Pathology, CEBAS-CSIC, E-30100, Murcia, Spain; b Institute of Plant Molecular and Cellular Biology (IBMCP CSIC–UPV), E-46022, Valencia, Spain; c de Duve Institute and MASSPROT Platform UCLouvain, 1200, Brussels, Belgium

**Keywords:** Abscisic acid, Oligomerization, Protein interaction, PYR1, Redox regulation, Thioredoxin *o*1

## Abstract

Abscisic acid (ABA) plays a fundamental role in plant growth and development processes such as seed germination, stomatal response or adaptation to stress, amongst others. Increases in the endogenous ABA content is recognized by specific receptors of the PYR/PYL/RCAR family that are coupled to a phosphorylation cascade targeting transcription factors and ion channels. Just like other receptors of the family, nuclear receptor PYR1 binds ABA and inhibits the activity of type 2C phosphatases (PP2Cs), thus avoiding the phosphatase-exerted inhibition on SnRK2 kinases, positive regulators which phosphorylate targets and trigger ABA signalling. Thioredoxins (TRXs) are key components of cellular redox homeostasis that regulate specific target proteins through a thiol-disulfide exchange, playing an essential role in redox homeostasis, cell survival, and growth. In higher plants, TRXs have been found in almost all cellular compartments, although its presence and role in nucleus has been less studied. In this work, affinity chromatography, Dot-blot, co-immunoprecipitation, and bimolecular fluorescence complementation assays allowed us to identify PYR1 as a new TRX*o*1 target in the nucleus. Studies on recombinant HisAtPYR1 oxidation-reduction with wild type and site-specific mutagenized forms showed that the receptor underwent redox regulation involving changes in the oligomeric state in which Cys^30^ and Cys^65^ residues were implied. TRX*o*1 was able to reduce previously-oxidized inactive PYR1, thus recovering its capacity to inhibit HAB1 phosphatase. *In vivo* PYR1 oligomerization was dependent on the redox state, and a differential pattern was detected in KO and over-expressing *Attrxo1* mutant plants grown in the presence of ABA compared to WT plants. Thus, our findings suggest the existence of a redox regulation of TRX*o*1 on PYR1 that may be relevant for ABA signalling and had not been described so far.

## Highlights

• PYR1 is a target of TRX*o*1 in the plant nucleus.
• PYR1 activity on HAB1 phosphatase is redox-regulated.
• TRX*o*1 is able to reduce oxidized PYR1 *in vitro* recovering lost activity.
• Oligomeric state of PYR1 is redox-dependent *in vitro* and *in vivo.*
• Lack or over-expression of *AtTRXo1* influence PYR1 oligomerization under ABA.

## Graphical abstract

**Figure:**
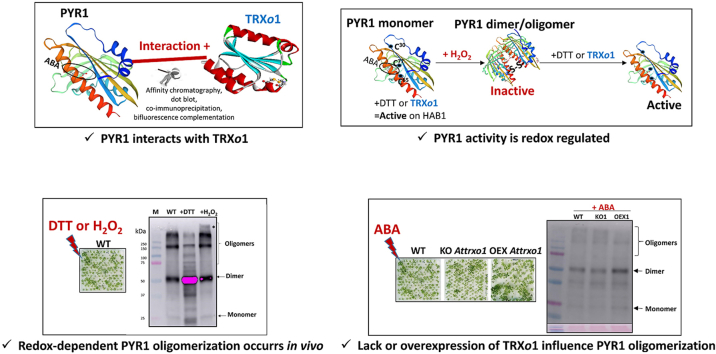

## Abbreviations

ABA: abscisic acid
ABI: ABA-insensitive
AOX: alternative oxidase
BiFC: bimolecular fluorescence complementation
BSA: bovine serum albumin
CoIP: co-immunoprecipitation
DMD: dimedone
DTT: 1,4-dithiothreitol
GFP: green fluorescent protein
HAB: hypersensitive to ABA
IPTG: isopropylthio-β-galactoside
KO: knock-out
MM(PEG)24: methyl-PEG24-maleimide
MS: mass spectrometry
NEM: n-ethylmaleimide
NTR: NADPH thioredoxin reductase
OEX: over-expressing
pNPP: p-nitrophenyl phosphate
PCNA: proliferating cell nuclear antigen
PP2C: type 2C phosphatase
PTM: post-translational modification
PRX: peroxiredoxin
PYL: PYR1-like
PYR: pyrabactin resistance
RCAR: regulatory component of ABA receptor 1
RNS: reactive nitrogen species
ROS: reactive oxygen species
RT-qPCR: reverse transcription-quantitative polymerase chain reaction
SnRK2: sucrose non-fermenting related protein kinase 2
TBS: Tris-buffered saline
TBSTA: Tris-buffered saline Tween albumin
TCEP: Tris(2-carboxyethyl)phosphine
TF: transcription factor
TRX*o*1: thioredoxin *o*1
WT: wild type

## 1 Introduction

Abscisic acid (ABA) plays a fundamental role in various aspects of plant growth and development, such as seed germination, stomatal response, adaptation to stresses, or fruit development and ripening [1,2]. In seeds, ABA regulates dormancy, longevity, and inhibition of germination under unfavourable conditions, like high salinity in the soil [3,4]. Developmental and stress-related conditions leading to the increase in endogenous ABA levels activate its signalling through specific receptors coupled to the function of several transducers. Ultimately, the transduced signal leads to the regulation of the expression of genes coding for proteins directly involved in the control of the hormonal imbalance and downstream actions. ABA signalling is orchestrated in plants through a core module comprising specific receptors, type 2C phosphatases (PP2C) and kinases of the sucrose non-fermenting 1-related -related protein kinase 2 (SnRK2) family, and ABA-responsive transcription factors and ion channels [5]. Soluble receptors belong to the PYR (pyrabactin resistance 1)/PYL (PYR1-like)/RCAR (regulatory component of ABA receptor 1) family [6,7]. After perception, the main transducers are phosphatases and kinases of the PP2C and SnRK2 families, respectively [8]. After the binding of ABA to receptors, the formation of a ternary complex with PP2Cs leads to the inhibition of their phosphatase activity, thus avoiding further de-phosphorylation of SnRK2s, which then remain active in phosphorylating diverse targets. PP2Cs, which are the main negative regulators of ABA signalling, can be subdivided into 11 classes in rice and Arabidopsis [9] and act as MAPK inhibitors [10]. Arabidopsis PP2Cs such as ABI1 and ABI2 (ABA-insensitive) or HAB1 and HAB2 (hypersensitive to ABA) regulate the response to ABA in a negative way [11] by inhibiting the kinase activity of three SnRK2s [12] that are required to phosphorylate transcription factors and ion channels propagating downstream signalling through transcriptional and non-transcriptional events.

The Arabidopsis family of ABA receptors can be classified in two groups: those forming dimers and those functioning as monomers. PYR1, PYL1, and PYL2 are *cis*-homodimers, whereas PYL3 also forms an intermediate *trans*-homodimer [13]. After ABA binding, these receptors undergo conformational changes that expose a binding surface for PP2Cs and cause the dissociation of PYL dimers, the formation of a PYL/PP2C complex, and the inhibition of phosphatase activity that release the PP2C-mediated inhibition of SnRK2 [5,14]. It has been described that ABA-bound PYL3 promoted the generation of monomeric PYL3 by increasing the inhibition of PP2Cs [13]. However, the exact mechanism, the importance of oligomerization on the PYR/PYL activity, and the involvement of redox regulation on these ABA receptors have not been elucidated. Post-translational modification (PTM) by S-nitrosylation, Tyr-nitration, and ubiquitination of several components of the ABA signalling pathway is an emerging topic to understand ABA-triggered responses [15,16]. Lately, redox regulation has also been described for some members of the PP2C family, such as ABI1 and ABI2 [17,18]. Interestingly, persulfidation of PYR/PYL receptors, as well as of some phosphatases and kinases, has been recently reported [19,20], suggesting that redox regulation on Cys residues could be a mechanism of control for ABA signalling. In fact, reportedly, modifications in thiol-containing enzymes under stress situations would be relevant in the regulation of their structure and functionality, thus suggesting their involvement in the perception of reactive oxygen and nitrogen species (ROS, RNS) [[21], [22], [23], [24]].

Thioredoxins (TRXs) are small redox proteins that reduce and activate oxidized target proteins, being consequently oxidized. TRXs are reduced back by NADPH-dependent thioredoxin reductase (NTR) in the different cell compartments, or by ferredoxin-dependent thioredoxin reductase (FTR) in chloroplasts. Among TRXs, the TRX*o*1 type has been located in mitochondria in Arabidopsis (Laloi et al., 2001) and also in nucleus in pea leaves (Martí et al., 2009), and several targets have been reported in both organelles, including some TCA-cycle enzymes, alternative oxidase (AOX), peroxiredoxin IIF in mitochondria, and proliferating cellular nuclear antigen (PCNA) in nucleus [[25], [26], [27]]. Nevertheless, its role in the nucleus has been far less studied. In this organelle, several redox-regulated transcription factors have been described, and some of them are targets of cytosolic thioredoxins or peroxiredoxins, provoking their translocation to the nucleus [22,28,29]. To deepen on the role of TRXs in the nucleus, in the present study, we have extended the previous information identifying PYR1 as a new TRX*o*1 target in this organelle by demonstrating their interaction by affinity chromatography, Dot-blot, co-immunoprecipitation, and bimolecular fluorescence complementation assays. Besides, *in vitro* experiments with recombinant proteins allowed us to propose that PYR1 oligomeric state and its effect on HAB1 phosphatase activity inhibition are modulated through a novel redox regulatory mechanism involving two out of three Cys residues of the receptor. *In planta*, our study revealed a differential pattern of PYR1 oligomerization in *Attrxo1* mutants versus WT plants grown in the presence of ABA.

## 2 Materials and methods

### 2.1 Identification of nuclear TRXo1 target proteins

The identification of nuclear target proteins from isolated nuclei of pea leaves was carried out as previously described [26] using DTT-reduced HisPsTRX*o*1C37S recombinant protein and a coaffinity resin (TALON Clontech, Takara Bio USA). Among the proteins that were revealed, several spots were excised from gels and analysed with a Proteineer DP (Bruker-Daltonics) in the Proteomic Service of the CNB–CSIC (Centro Nacional de Biotecnología, Madrid, Spain). After MS/MS analysis, data were analysed with the Mascot algorithm (Matrix Science, UK) and revised using the BLAST protein program (www.expasy.org).

### 2.2 Dot-blot assay

Five drops of equal volume (10 μl), each containing 1 μg or 0.5 μg of PsTRX*o*1 and HisPsTRX*o*1C37S (active centre mutated in Cys^37^ by a Ser) [25] recombinant proteins, or 10 μg of BSA (bovine serum albumin, Sigma-Aldrich) as a negative control, were blotted onto two nitrocellulose membrane strips (Hybond, Amersham Pharmacia, United Kingdom). After a 5-min wash with TBS (25 mM Tris-HCl, pH 7.5), nitrocellulose strips were blocked by incubation in TBSTL buffer (25 mM Tris-HCl, pH 7.5 containing 5% [w/v] Hero Baby Nutrasense non-fat dry milk powder and 0.1% [v/v] Tween-20) or TBSTA buffer (25 mM Tris-HCl, pH 7.5 containing 1% [w/v] BSA and 0.1% [v/v] Tween-20) for 1 h at room temperature. Then, membranes were covered with 10 μg/ml of recombinant HisAtPYR1 protein dissolved in TBS and incubated for 2 h at room temperature. After washings, membrane A was treated with the polyclonal anti-PYR1 (1:10,000 in TBSTL, Agrisera) antibody and membrane B was incubated with polyclonal anti-PsTRX*o*1 (1:2,000 in TBSTA, Abyntek, Vizcaya, Spain). After thorough washing with TBST, membranes were incubated with the secondary anti-rabbit antibody coupled to horseradish peroxidase (1:30,000 in TBSTA, Merck KGaA, Darmstadt, Germany). The PIERCE ECL 2 kit (ThermoScientific) was used for protein visualization by chemiluminescence.

### 2.3 Co-immunoprecipitation of PsTRXo1 and HisAtPYR1

Co-immunoprecipitation of the PsTRX*o*1 and HisAtPYR1 proteins was analysed using the commercial μMACS Anti-His magnetic MicroBeads kit (MACS Miltenyi Biotec). HisAtPYR1/PsTRX*o*1 and HisAtPYR1/PsTRX*o*1C37S protein combinations (4 μg of each protein) were diluted with 500 μL of lysis buffer provided with the kit (50 mM Tris-HCl, pH 8.0 containing 150 mM CaCl_2_). Previously, thrombin was used to cleave the His from the mutated version of the HisTRX*o*1C37S protein using the Thrombin CleanCleave™ Kit (SIGMA) and following the manufacturer's instructions. After removing 50 μL of the diluted proteins for further use as a loading control, the remaining proteins were treated with 50 μL of the anti-His μbead suspension and incubated for 2 h at 4 °C under continuous rotary shaking. After magnetic columns conditioning with 200 μL of lysis buffer, the suspension of proteins and magnetic beads was loaded into the columns and washed four times with 200 μL of wash buffer 1 (150 mM NaCl, 1% Igepal CA-630, 0.5% sodium deoxycholate, 0.1% SDS, 50 mM Tris-HCl, pH 8.0). Then, columns were rinsed with 100 μL of wash buffer 2 (20 mM Tris-HCl, pH 7.5). Proteins were eluted from the columns, after a 5-min pre-incubation at room temperature with 20 μL of elution buffer (50 mM Tris HCl, pH 6.8, 50 mM DTT, 1% SDS, 1 mM EDTA, 0.005% bromophenol blue, 10% glycerol) preheated to 95 °C, by loading four consecutive 50 μL treatments with preheated elution buffer at 95 °C. The eluate was analysed by SDS-PAGE on AnykD gels (Bio-Rad, Spain) and Western blot as previously described [30]. Polyclonal antibody for PsTRX*o*1 was used as described above. Monoclonal mouse anti-His (1:5000 in TBSTA, Agrisera) as primary antibody and anti-mouse coupled to horseradish peroxidase (1:10,000 in TBSTA, ABCAM) as secondary antibody were used for PYR1 detection. A control experiment using anti-His on a spotted thrombin-treated HisPsTRX*o*1C37S and AtPYR1 proteins (as a positive control) was carried out to ensure the absence of His in the recombinant mutated TRX*o*1 protein, using serial dilutions of 0.4 mg/ml aliquots. The PIERCE ECL 2 kit (ThermoScientific) was used for protein visualization.

### 2.4 Bimolecular fluorescent complementation (BiFC) in Nicotiana benthamiana

**Fig. S1:**
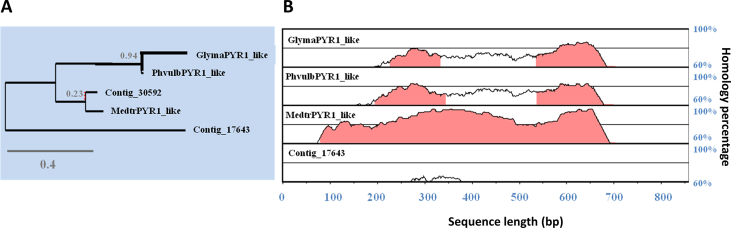
(A) Phylogenetic tree of PYR1 orthologues in legumes. It was done using phylogeny.lirmm.fr/phylo_cgi/. Accession number in Phytozome database: *Glycine maxima* (Glyma.01g097000); *Phaseolus vulgaris* (Phvul.003G285500); *Medicago truncatula* (Medtr5G030500). (B) Mapping of conserved sequences (pink >70% homology among PYR orthologues) was done using: http//genome.lbl.gov/vista/mvista/submit.shtml.

**Fig. S2:**
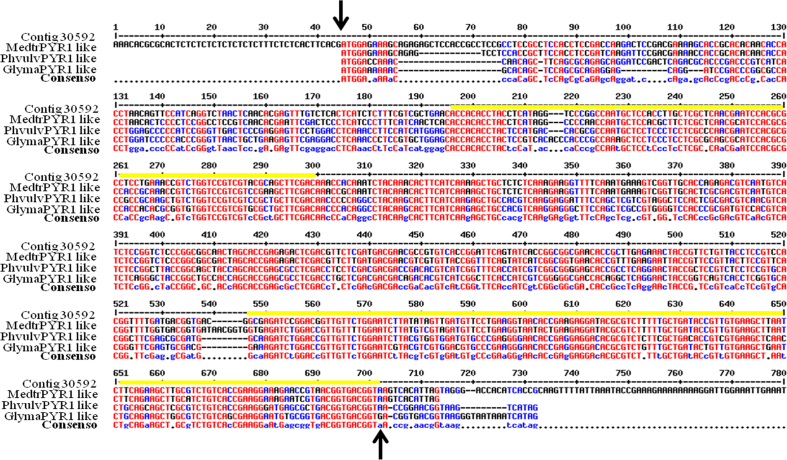
Sequence alignment among PYR1 orthologues. In red, the conserved areas of the 4 genes; in blue, only those of 3 genes; and in black, less than 2 genes. Yellow colour indicates the most conserved areas with >70% homology among the orthologues identified with the *mvista* program. Arrows indicate the most probable start and end of the PYR1 ORF. Done using: tttp//multalin.toulouse.inra.fr/multalin.

The sequences for *AtTRXo1*, *AtPYR1,* and *PsTRXo1* were obtained from TAIR (NM_129053, NM_117896) and EMBL (Q257C6) databases. For the PsPYR1 sequence, we conducted an *in silico* analysis of contig sequences of leguminous plants (TBLASTn and *Pisum sativum* unigene Franssen database: https//www.coolseasonfoodlegume.org) using the two peptides obtained after the affinity chromatography with the mutated TRX*o*1 on pea extracts (see results on section 3.1). These peptides were described as possible PYR1 peptides compared to PYR1 sequences of different leguminous species (Figs. S1A and S1B). From the pea database, two contigs (30592 and 1793) showing more than 50% of homology with the bait sequence were chosen. A phylogenetic tree with the leguminous sequences allowed us to choose contig 30592 as more similar to the *Medicago truncatula* sequence, and after using the mVISTA and Multalin programs, we discriminated the extension and possible sequence of the PsPYR1 ORF (Fig. S2). The complete sequence was obtained with the 3′-5′ RACE technique [31], using the oligonucleotides Q_t_ adapter, Q_0_-tailS1, and Q_1_-tailS2 for the 3′ extreme (Table S1, primers 1–3). After synthesizing cDNA from pea RNA, three nested-PCR were performed using Q_0_-GSP1, Q_1_-NGSP1, and Q_1_-NNGSP1 primers (Table S1, 4–6). For the 5′ extreme, we used the oligonucleotide RT-GSP 5′ RACE (antisense of a conserved region of the gen, primer 7), and, after adding deoxy-CTP to synthetized cDNA with the terminal deoxynucleotidil transferase (TdT) enzyme, we used it for the first complementation with the UAP-adapter (primer 8) followed by 3 nested-PCR using Q_0_-GSP2, Q_1_-NGSP2 and, finally, Q_1_-NNGSP2 primers (Table S1, primers 9–11). Once the 3′ and 5′ ORF were obtained, the complete gene was amplified and cloned into pGEM-Teasy vector. The LR reaction of the Gateway technique was used to fuse part of the Green Fluorescent Protein (GFP) with the ORFs of *AtTRXo1*, *PsTRXo1*, *AtPYR1,* and *PsPYR1* genes in the destination vectors pNX (N-terminal domain of *GFP* to be cloned in the N-terminal of *TRXo1/PYR1*), pXN (N-terminal domain of *GFP* to be cloned in the C-terminal of *TRXo1/PYR1*), pCX (C-terminal domain of *GFP* to be cloned in the N-terminal of *TRXo1/PYR1*), and pXC (C-terminal domain of *GFP* to be cloned in the C-terminal of *TRXo1/PYR1*) as previously described [32,33] (Table S1, primers 12–31). The generated constructs under the 35S promoter were: *pAtTRXo1-CGFP*; *pAtTRXo1-NGFP*; *pCGFP-AtTRXo1*; *pNGFP-AtTRXo1*; *pPsTRXo1-CGFP*; *pPsTRXo1-NGFP*; *pCGFP-PsTRXo1*; *pNGFP-PsTRXo1*; *pAtPYR1-CGFP*; *pAtPYR1-NGFP*; *pCGFP-AtPYR1*; *pNGFP-AtPYR1*; *pPsPYR1-CGFP*; *pPsPYR1-NGFP*; *pCGFP-PsPYR1*; *pNGFP-PsPYR1*. *Agrobacterium tumefaciens* was transformed with the different constructs by electroporation and used to infect *Nicotiana benthamiana* plants. *ATML1* and *PDF2* cloned in the same vectors were used as positive controls to set up the parameters for the microscopy analysis [32]. The amino- and carboxy-terminal GFP constructs were used as negative controls co-expressed with the complementary version ORF GFP fused to *TRXo1* or *PYR1*. The resulting constructs were used to genetically transform *Agrobacterium tumefaciens* C58C1 GV3101 by electroporation and subsequent selection on SOB medium supplemented with 50 mg/L of rifampicin and spectinomycin, as well as 40 mg/L of gentamicin. Two days before agroinfiltration of *Nicotiana benthamiana* plants, they were acclimated in a Sanyo culture chamber (MLR-351 model in long-day photoperiod: 16 h of light:8 h of darkness, 22 °C and 20 °C, respectively with a light intensity of 150 μEm^−2^s^−1^ by cold white fluorescent lamps and 60% relative humidity). Different lines of *Agrobacterium tumefaciens* previously transformed with the different constructs, as well as a *p19* plasmid to inhibit possible gene silencing by the host plant, were incubated for 3 h in an induction solution (10 mM MgCl_2_, 10 mM MES [2-(N-morpholino acid)], 100 μM acetosyringone) with an OD_600_ = 3 to activate its *vir* genes as previously described [34]. Subsequently, each bacterial line was agroinfiltrated with a 1:1:1 (*p-PsTRXo1:p-AtPYR1:p19*) ratio at OD_600_ = 0.3 in leaves of *Nicotiana benthamiana* plants with a 1 ml syringe.

### 2.5 Confocal microscopy

After 72 h from the agroinfiltration of *Nicotiana benthamiana* leaves, leaf discs were punched by using an 8-mm-diameter cork borer. Fluorescence in the leaf disks was analysed using a Zeiss LSM 780 confocal microscope equipped with an Apochromat 40X/1.2W lens. Zooms of 0.6 and 1.2 were applied to the general images, and a zoom of 2.4 for details. For the fluorescence detection, excitation was performed at 488 nm 2% transmission (MBS; dichroic 488), and the emission was detected in the range of 495–536 nm (gain of PMT to 900) for the GFP and in the range of 684–758 nm (gain of PMT at 708) for chlorophyll. Software used to capture images was Zen 2011.

### 2.6 Expression and purification of recombinant mutant PYR1 proteins

The different fragments of mutated residues in the PYR1 sequence (At4g17870 TAIR) in which cysteine residues Cys^30^, Cys^65^, and Cys^77^ were replaced by serine were obtained by reverse transcription-PCR. These fragments were cloned into the *Nco*I/*EcoR*I sites of the pETM11 expression vector which adds a His tail, following Castillo et al. [15] (Table S1, primers 32–39). Constructs were verified by sequencing. *Escherichia coli* BL21 strains were transformed with the resulting constructs, and recombinant protein expression was induced by the addition of 1 mM isopropylthio-β-galactoside (IPTG) for 3 h at 37 °C. The purification of the recombinant AtPYR1 mutated His-tag proteins was performed using a nickel affinity chromatography system following the manufacturer's instructions (Ni-NTA Agarose, Qiagen).

### 2.7 PYR1 activity

PYR1 activity was colorimetrically measured in a coupled assay to phosphatase HAB1 activity that dephosphorylates the substrate p-nitrophenyl phosphate (pNPP), giving rise to the intermediate product p-nitrophenol, which under alkaline conditions generates p-nitrophenolate, a coloured final product, which allows the reaction to be monitored by measuring the increase of the absorbance at 405 nm as previously described [15]. The presence of PYR1 and ABA decreases the speed of the reaction by inhibiting phosphatase, and that decrease is due to PYR1 activity as ABA receptor.

For the tests with reduced or oxidized PsTRX*o*1 and HisAtPYR1 proteins, previous treatments were elaborated by slightly modifying the method followed by Calderón et al. [26]. Between 100 and 120 μg of recombinant protein were dissolved in a final volume of 500 μL of 20 mM Tris-HCl buffer, pH 7.9 containing 50 mM NaCl and 5% glycerol (v/v). In the case of reduced proteins, 2 mM DTT was added to the dilution and shaked by inversion at 4 °C for 1 h. To oxidize proteins, 4 mM H_2_O_2_ was added and incubated for 1 h at room temperature. After reduction or oxidation treatments, the 500 μL with modified proteins were filtered by centrifugation through Amicon™ Ultra-0.5 mL 3K devices. Then, 400 μL of the new buffer without DTT or H_2_O_2_ were loaded and centrifuged again. This process was repeated twice to finally collect the concentrated proteins without DTT or H_2_O_2_.

For measuring phosphatase activity, a molar ratio of 1:2 (phosphatase:PYR1) was used and, when indicated, 2 μM PsTRX*o*1, previously reduced by DTT as described above, was added. Proteins were incubated for 10 min at room temperature in Milli-Q water in the absence (−) and presence (+) of 10 μM ABA. The reaction took place in 100 μL of reaction solution containing 25 mM Tris-HCl, pH 7.5, 2 mM MnCl_2_, and 10 mM pNPP (Sigma-Aldrich), measuring every 30 s during 30 min in a microplate spectrophotometer (Multiskan GO, Thermoscientific).

### 2.8 Analysis of the redox state of PYR1

PsTRX*o*1 and HisAtPYR1 recombinant proteins previously modified by oxidation or reduction used for PYR1 activity assays were subjected to Western blot analysis. For this purpose, the same incubation conditions as those applied in the PYR1 activity tests were used. After SDS-PAGE electrophoresis, proteins were transferred to nitrocellulose membranes in transfer buffer (48 mM Tris-HCl, pH 8.9 containing 39 mM Glycine, 0.03% SDS [w/v] and 20% methanol [v/v]) by applying an electric field with a constant voltage of 25 V and 2.5 mA for 10–15 min using a semi-dry transfer device (Trans Blot Trubo®, Bio-RadTM). Membrane was blocked by incubation for 1 h at room temperature with 3% (w/v) BSA in TBS. After this step, membrane was incubated with the specific primary antibody diluted in TBS overnight at 4 °C. Membrane was washed in TBST and incubated for 1 h at room temperature with HRP-conjugated secondary antibody diluted in TBS. After washing with TBST, Super Sigma west Pico Plus kit (Thermofisher) was used to detect proteins. Densitometry of the different bands was performed using an image analyser (Amersham Imager 600, GE Healthcare, Barcelona, Spain) and the Amersham ImageQuanTL 8.1 Program (Cytiva, Barcelona, Spain). Ponceau staining allowed us to check the loading.

Recombinant HisAtPYR1 was also used to perform redox treatments like that described in Calderon et al. [26] with some modifications. For that, 50 μg of protein were dissolved in 500 μL of reaction buffer 20 mM Tris-HCl, pH 7.9. For reduced protein, 2 mM DTT was added and incubated during 30 min at room temperature. For oxidative treatment, protein was incubated with 4 mM H_2_O_2_ during 30 min at room temperature. Another sample was previously treated with 2 mM DTT as described above, and the excess of DTT was eliminated using Amicon filters (by centrifuging during 30 min at 14,000×*g* at 4 °C) and then oxidized with 4 mM H_2_O_2_ as described above. 2.5 μg of treated samples were subjected to SDS-PAGE and Western blot using anti-AtPYR1 antibody. When using the TRX system as a reductant instead of DTT, previously reduced PYR1 protein with 2 mM DTT was oxidized with H_2_O_2_ as described above, and then 3 μg of protein were incubated in Tris-HCl 50 mM pH 7.5 buffer containing 10.7 μM TRX*o*1, 0.3 μM NTRB, and 2.8 mM NADPH for 1 h at 30 °C. A control with NADPH and NTRB without TRX*o*1 was also performed.

For mass spectrometry analysis, recombinant PYR1 protein was reduced by 2 mM DTT (reduced PYR1) or DTT-treated followed by filtration and treated with 4 mM H_2_O_2_ (oxidized PYR1) as described above. Another sample was DTT-reduced before being incubated with 2 mM dimedone (5,5-dimethyl-1,3-cyclohexanedione) and 4 mM H_2_O_2_ together to label the possible sulfenic state of the Cys residues. All of them were finally treated with 4 mM NEM (in 50 mM Tris-HCl, pH 7.5 buffer) for 10 min at RT to label the free –SH groups. Recombinant proteins were then precipitated with 15% TCA and washed twice with 1 mL 80% cold acetone, and pellets were allowed to dry. They were resuspended in 40 μL of 100 mM ammonium bicarbonate pH 8.0 and digested with trypsin, as described before [35]. Samples were analysed by nano LC-MS on an Orbitrap Fusion Lumos with a data dependent scan routine/and charge state selection of the fragmentation mode: CID:IT for +2 and + 3 ions or EThcD:OT for +4 and higher Raw MS data were processed with the Proteome Discoverer 2.5 program. Disulfide pattern was analysed with the pLink2 program and manually validated.

The existence of S–S bridges was investigated in the recombinant protein by a redox mobility shift assay, HisAtPYR1 was alkylated as previously described for PCNA protein with some modifications [26]. Recombinant AtPYR1 protein (80 μg) was previously reduced with 2 mM DTT in 100 μL of reaction buffer 50 mM Tris-HCl, pH 7.5 for 30 min at room temperature. After eliminating the excess of DTT, 50 μg of DTT-treated recombinant protein were oxidized in a total volume of 50 μL with 4 mM H_2_O_2_ for 1 h at room temperature in the reaction buffer and dialyzed using the same chromatography columns. Reduced and oxidized AtPYR1 proteins (5 μg) were incubated with 10 mM methyl-PEG24-maleimide (MM[PEG]24, ThermoScientific Pierce) for 20 min at RT in darkness and then analysed in AnykD gels (Bio-Rad, Spain) by non-reducing SDS-PAGE and Western blot.

### 2.9 Plant material

Seeds of *Arabidopsis thaliana* ecotype Columbia (Col-0; wildtype, WT) and the T-DNA insertion knock-out mutants *AtTRXo1* SALK_143294C (KO1) and SALK_042792 (KO2) were grown on plates as previously described [27]. We also analysed two stable transformed lines of *A. thaliana* overexpressing the *AtTRXo1* gene, which were generated as described below. For ABA treatment experiments, seedlings grown for 14 days were transferred to plates containing 10 μM ABA and grown for 48 h.

#### 2.9.1 Generation of AtTRXo1-overexpressing lines

Total RNA was extracted from *A. thaliana* leaves using the RNeasy Plant Mini Kit (Qiagen, Germany) and reverse transcription was performed with 2 μg of total RNA pre-treated with DNase I (Roche, Germany) to obtain cDNA using the High-capacity cDNA Reverse Transcription Kit (Applied Biosystems, Spain). The ORF of the *AtTRXo1* gene was amplified using the specific oligonucleotides *AtTRXo1for-AtTRXo1rev* in a first PCR event, followed by a second one using *AttB1tail5′-AttB2tail3*’ primers (Table S1, primers 40–43) for a nested-PCR that incorporated the recombination tails needed to clone the insert in both vectors with Gateway technology. DNA was then cloned into the pDNOR221 vector using BP clonase enzyme from Invitrogen as recommended by the manufacturer's instructions. Using Gateway technology, the gene was subsequently cloned into the pMDC32 target vector by recombination reaction using LR clonase enzyme. These constructs were used to transform competent strains of *E. coli*, and the corresponding plasmids were verified by sequencing and subsequently used to transform competent strains of *A. tumefaciens*. The transformed bacteria were selected by plating with the antibiotics rifampicin (Rf), gentamicin (Gm), and kanamycin (Km) for 36 h at 28 °C in darkness and with 120-rpm orbital agitation. One of the colonies that were obtained was cultured in 10 mL of selective culture medium (sterile LB with the corresponding antibiotics, 1% tryptone [w/v]; 1% NaCl [w/v]; 0.5% yeast extract [w/v], pH 7.0), under appropriate conditions for 24 h. This pre-inoculum was added to 250 mL of selective culture medium and incubated under appropriate conditions for 24 h. When the cell culture reached the exponential phase of growth, 200 μL of Silwet L-77 (Lehle seeds) detergent was added.

For the plant transformation and selection, infiltration of *A. tumefaciens* was carried out in 18-cm inflorescences of plants containing only closed flower buds according to Zhang et al. [36]. Immersion of the inflorescence in the bacterial culture occurred for 50 s. The excess infiltration solution was removed by placing the plants on filter paper. Plants were placed horizontally in a tray covered with a perforated plastic film and left in a culture chamber at 22 °C–18 °C (day night), with a light intensity of 135 μmol m^−2^ s^−1^ (PAR), 60% relative humidity, and long day photoperiod 16:8 h. After 24 h, plastic was removed, placed in an upright position, and watered until it was used for seed collection. The selection of the *A. thaliana* mutant lines was carried out in 9 × 9 cm Petri dishes on solid medium supplemented with the resistance antibiotic hygromycin. T2 populations with 75% seeds resistant to hygromycin were selected for further isolation of T3 homozygous over-expressing (OEX) lines. Two OEX lines (OEX1 and OEX2) were chosen for further analysis.

#### 2.9.2 Analysis of TRXo1 overexpression in OEX mutants

##### 2.9.2.1 Quantitative RT-PCR (qRT-PCR)

To check *AtTRXo1* expression, total RNA was obtained from rosette leaves of two homozygous over-expressing lines. *AtTRXo1* expression level was estimated by qRT-PCR (Illumina) in the Molecular Biology Services at the University of Murcia, as previously described [37] using the primers *AtTRXo1for*, *AtTRXo1rev*, and, for the housekeeping gen *Actin8*, *AtActin8for* and *AtActin8rev* (Table S1, primers 44–47). Five biological replicates were analysed with their respective technical replicates. Each biological replicate consisted of groups of 10 seedlings.

##### 2.9.2.2 Western blot analysis

Mitochondria were isolated by differential and density Percoll gradient centrifugation from rosette leaves of Arabidopsis plants grown in Murashige and Skoog plates as previously described [27]. Mitochondrial proteins were solubilized with Triton X-100 0.1% (v/v) and incubated 30 min at 4 °C. After centrifugation at 14,000×*g* for 10 min, the supernatant was boiled for 5 min in Laemli buffer containing 50 mM DTT. Then, proteins were separated by SDS-PAGE. 4 μg of recombinant HisPsTRX*o*1 were used as a positive control and 100 μg of mitochondrial proteins were used in each lane and subjected to Western blot as described above using anti-AtTRX*o*1 antibody.

Nuclei were isolated from frozen Arabidopsis rosette leaves that were homogenized in liquid nitrogen, resuspended in cold 0.5 M PIPES (piperazine-N-N′-bis-2-ethanesulfonic acid), pH 7.0 containing 1 M hexylene glycol, 10 mM MgCl_2_, and 1 mM DTT, and then passed through two layers of Miracloth as described in Bae et al. [38]. All of the following steps were carried out on ice or at 4 °C following Liu et al. [39] with minor modifications. Briefly, the homogenate was centrifuged at 1500×*g* for 5 min and the pellet was resuspended in glycerol buffer (20 mM Tris-HCl, pH 7.9 containing 50% glycerol, 75 mM NaCl, 0.5 mM EDTA, 0.85 mM DTT, 0.125 mM PMSF and protease inhibitor cocktail from Roche). The same volume of prechilled nuclei lysis buffer (10 mM HEPES, pH 7.6, 1 mM DTT, 7.5 mM MgCl_2_, 0.2 mM EDTA, 0.3 M NaCl, 1 M urea, 1% NP-40, 0.5 mM PMSF, 10 mM β-mercaptoethanol, and Roche protease inhibitor cocktail) was then added, and the mixture was vortexed, incubated for 2 min on ice, and centrifuged at 14,000 rpm for 2 min. Supernatant was collected as nucleoplasmic fraction, and proteins were measured by Bradford's method [40] before being analysed by Western blot.

### 2.10 PYR1 oligomerization pattern in plant extracts

PYR1 oligomerization pattern was analysed in leaf extracts homogenized in lysis buffer consisting of 50 mM Tris-HCl, pH 7.5, 0.5% Triton X-100, 150 mM NaCl, 1 mM PMSF, and protease inhibitors cocktail (Roche, Spain) containing 5 mM N-ethylmaleimide (NEM) to block all of the free cysteine thiols. Extracts were incubated for 30 min in darkness at room temperature and gentle agitation, and then lysates were centrifuged at 15,000 g for 5 min at 4 °C. Soluble proteins were quantified using a 1:5 dilution to avoid interferences by the detergent and 5 μg (for a better visualization of the dimer) and 15 μg (for visualization of the monomer and oligomers) were subjected to SDS-PAGE and Western blot in AnykD gels (Bio-Rad, Spain) using anti-PYR1 (1:10,000, Agrisera, Spain).

To check the effect of reductive and oxidative treatments on leaf extracts of WT plants, extracts were incubated in the presence of 50 mM DTT for 1 h at 4 °C, or with 4 mM H_2_O_2_ for 1 h at room temperature to be further analysed by Western blot. To identify the presence of disulfide (S–S) groups in the different oligomeric forms of PYR1, leaf extracts, obtained in the presence of NEM as described above, were desalted and concentrated with Amicon (Millipore, Spain) filters to eliminate the remaining NEM reagent, and then treated with 5 mM Tris(2-carboxyethyl)phosphine (TCEP) at room temperature for 30 min to reduce all of the cysteine disulfides. Recovered cysteine thiols were modified by reaction with 6.25 mM MM[PEG]24 at room temperature for 4 h, which added around 12,000 Da label to the free thiols. Modified proteins were desalted again on Amicon filters. Labelled proteins were analysed by Western blot after non-reducing SDS-PAGE in AnykD gels (Bio-Rad).

### 2.11 Statistical analysis

Experiments were conducted in a completely randomized design. The results that are presented here are the mean of at least three biological replicates from each experiment, and all of the experiments were repeated at least three times. Data were subjected to a Student's t-test (* at P < 0.05) using the IBM SPSS Statistics program (Statistical Package for Social Sciences, 2011).

## 3 Results

### 3.1 Analysis of the interaction PYR1-PsTRXo1

**Fig. 1:**
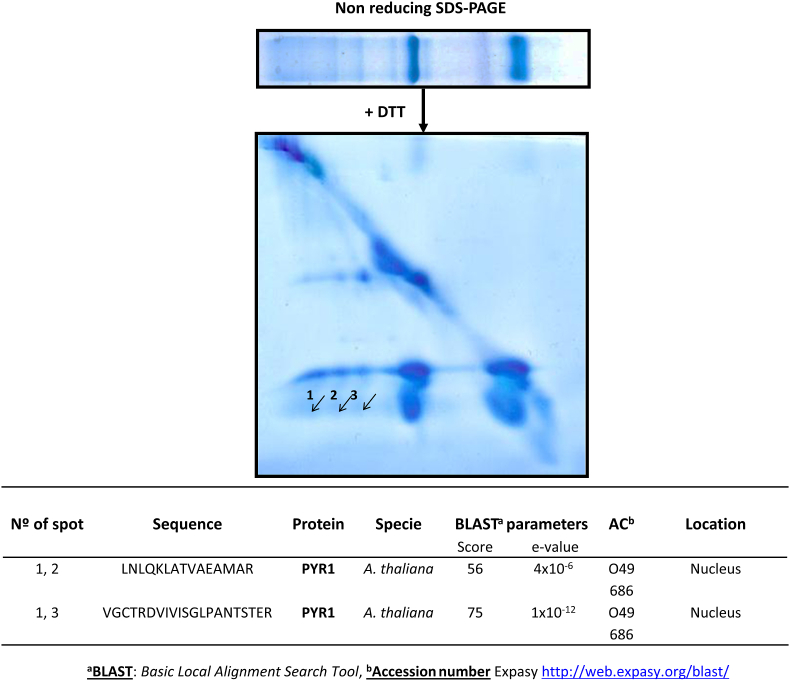
Protein pattern of pea nuclear target proteins of HisPsTRXo1C37S. Affinity chromatography and 2D SDS-PAGE in non-reducing (1st dimension) and reducing (2nd dimension) conditions of the eluted complexes. Gels were stained with Blue Page^R^. Arrows are drawn on the spots in which the peptides corresponding to PYR1 (pyrabactin resistance 1) were identified by MALDI MS/MS sequencing as shown in the table. Data were combined using the BioTools program (Bruker-Daltonics) to search in the non-redundant databases (NCBInr and SwissProt) with the Mascot (Matrix Science, UK) software. All data were manually revised using the BLAST protein program (www.expasy.org). (For interpretation of the references to colour in this figure legend, the reader is referred to the Web version of this article.)

To identify the potential nuclear targets of PsTRX*o*1, we used a mutant variant of PsTRX*o*1 in which Cys^37^ is substituted by a serine (HisPsTRX*o*1C37S) to stabilize TRX*o*1-target protein interactions, and performed an affinity chromatography assay on a His-binding co-affinity resin column using nuclear proteins. For that, we obtained nuclei from pea leaves, as previously done for the identification of the proliferating cellular nuclear antigen (PCNA) target protein [26]. Then, proteins were subjected to two-dimensional electrophoresis, being the first dimension done under non-reducing conditions, followed by the second one under reducing conditions. Obtained spots were then analysed by mass spectrometry after staining with Blue Page reagent. BLAST of the MS data corresponding to three of the stained spots (Fig. 1) did not match with any sequence in the still scarcely known pea genome, but allowed us to assign a closest match to the *Arabidopsis thaliana* ABA receptor PYR1 (pyrabactin-resistance 1).

**Fig. 2:**
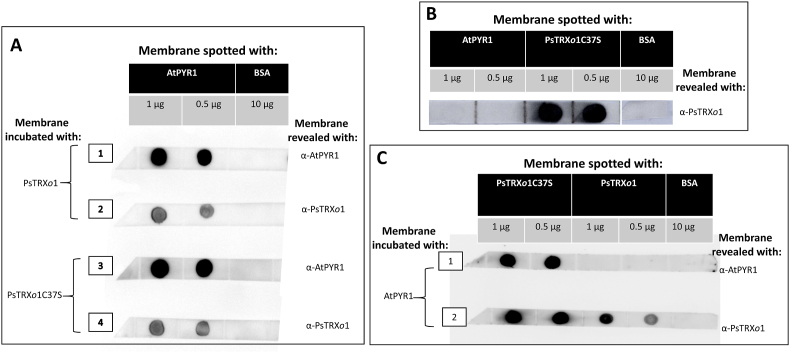
(A) Protein gel Dot-blot analysis of the interaction between recombinant AtPYR1 and PsTRXo1/PsTRXo1C37S. Membranes were spotted with AtPYR1 and BSA and were overlaid with TBS buffer containing 10 μg/mL recombinant PsTRX*o*1 or PsTRX*o*1C37S. The presence of the proteins was revealed using anti-AtPYR1 and anti-PsTRX*o*1 antibodies. BSA was spotted onto the membranes as a negative control. (B) Protein gel Dot-blot analysis for the detection of PsTRX*o*1C37S protein and not AtPYR1 or BSA by the anti α-TRX*o*1 antibody. (C) Protein gel Dot-blot analysis of the interaction between recombinant AtPYR1 and PsTRX*o*1C37 or PsTRX*o*1. Membranes were spotted with PsTRX*o*1C37S, PsTRX*o*1, and BSA (as a negative control) and were overlaid with TBS buffer containing recombinant AtPYR1. The presence of the proteins was revealed using anti-AtPYR1 and anti-PsTRX*o*1 antibodies.

To check whether PYR1 was a PsTRX*o*1 target, we decided to verify the interaction in pea and Arabidopsis. We first carried out a Dot-blot analysis of the interaction between purified recombinant HisAtPYR1 and two versions of PsTRX*o*1, the wild type and the mutant HisPsTRX*o*1C37S protein, which may display a higher stabilization rate with target proteins. These proteins were spotted onto nitrocellulose membranes, washed, blocked with non-fat dry milk powder, and incubated with TBS solution containing the protein with a potential to interact. The presence of both interacting proteins (PYR-TRX*o*1 or PYR1-TRX*o*1C37) on the nitrocellulose strips was revealed by using specific primary antibodies of both the immobilized protein (positive control) and the mobile protein that could be retained by the immobilized protein, which allowed for an interaction checking. For the negative control, albumin (BSA) was used as an immobilized protein. The presence of TRX*o*1 and TRX*o*1C37S bound to spotted PYR1 on the membrane was demonstrated by using anti-TRX*o*1 antibody (Fig. 2A, lanes 2 and 4), whereas the presence in these fractions of the spotted PYR1 was checked with an anti-PYR1 antibody (Fig. 2A, lanes 1 and 3). The control of detection of PsTRX*o*1C37, and not PYR1, by the anti-TRX*o*1 antibody is presented in Fig. 2B.

We also carried out the experiment with spotted PsTRX*o*1 and PsTRX*o*1C37S, incubating with recombinant AtPYR1 as a mobile protein and revealing with both anti-AtPYR1 (Fig. 2C, lane 1) and anti-PsTRX*o*1 (as a positive control, Fig. 2C, lane 2). In this case, the presence of PYR1 was positive only when the mutated TRX*o*1C37S was spotted, which suggests that the mutated version of TRX*o*1 helped stabilizing the interaction with the receptor. This assay also corroborated the interaction between PYR1 and PsTRX*o*1, which was specific, since no signal was detected in positions corresponding to equivalent amounts of BSA. It also confirmed that no cross-reaction between the antibodies existed.

**Fig. 3:**
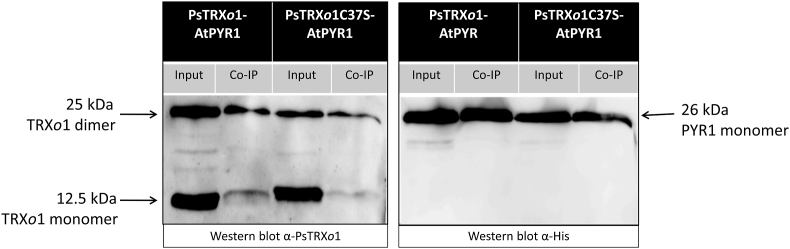
Western blot of the resulting Co-IP. The presence of wild type and mutated PsTRX*o*1 versions and AtPYR1 recombinant proteins was revealed by Western blot with anti-PsTRX*o*1 and anti-polyhistidine antibodies. The product band of PsTRX*o*1 recognition on the PsTRX*o*1 monomer was displayed at 12.5 kDa. The 25 kDa band on the membrane incubated with anti-PsTRX*o*1 corresponded in molecular weight to the dimer of PsTRX*o*1, whereas on the membrane incubated with anti-His, the 25 kDa AtPYR1-His protein was detected.

**Fig. S3:**
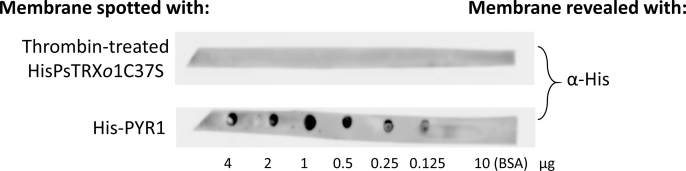
Protein gel dot-blot analysis for the detection of His tag in thrombin-treated HisPsTRX*o*1C37S and HisPYR1 proteins by the anti α-His antibody. BSA (10 μg) was loaded as a negative control.

The immobilization of one of the proteins might introduce a bias in the interaction analysis; hence, we confirmed the interaction in liquid phase by a co-immunoprecipitation (Co-IP) assay with recombinant proteins. After incubation of HisAtPYR1 with any of the two versions of PsTRX*o*1, being the mutated protein previously treated with thrombin to eliminate the His tag (Fig. S3), anti-His antibody-coated magnetic beads were added to the protein mixture. After incubation at 4 °C, the mixture was loaded onto a magnetic column and extensively washed, and HisAtPYR1 protein together with interacting proteins were eluted and analysed by Western blot. Fig. 3 presents in the first and third lanes the “input” equivalent to a fraction of the solution in which proteins were incubated before passing through the column, while the second and fourth lanes show the content of the eluate with the retained proteins. As can be seen, protein PsTRX*o*1 was obtained in the eluate in its two versions, as a monomeric form of 12.5 kDa and the dimer of 25 kDa, together with the 25 kDa protein HisAtPYR1 evidenced in the same membrane after mild stripping and subsequent Western blot with anti-His. These data confirm the interaction between HisAtPYR1 and both PsTRX*o*1 and PsTRX*o*1C37S proteins.

**Fig. 4:**
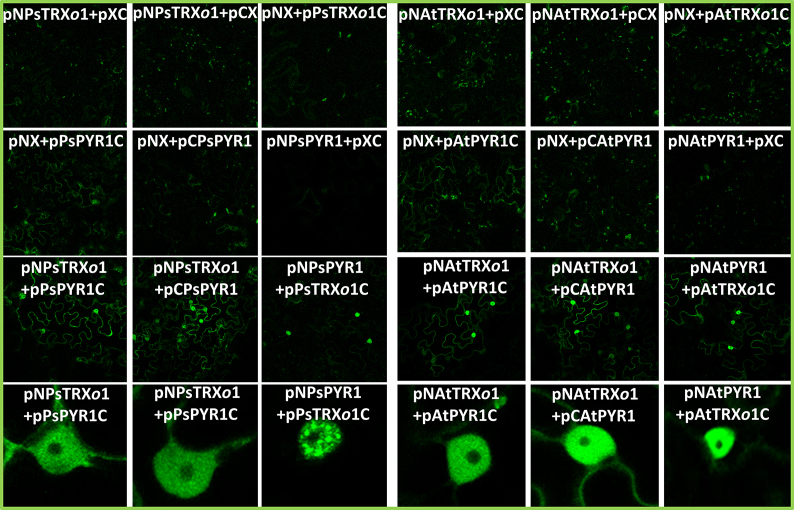
BiFC assay in *Nicotiana benthamiana* with combinations of Arabidopsis and pea proteins. Positive results of the interaction TRX*o*1-PYR1 after Agrobacterium transformation of *Nicotiana benthamiana* with the different combinations of constructs of the pea and Arabidopsis genes, cloned into pCX, pXC, pNX, and pXN vectors. Letters N and C indicate the presence of the N-terminal or C-terminal extreme of the GFP in the vector. X represents the relative position of the gene of interest related to the GFP half in which it is fused. Last panels at the bottom present vesicle-associated GFP reconstitution in some TRX*o*1-PYR1 BiFC assays.

The *in planta* interaction between TRX*o*1 and PYR1 was also analysed by means of a Bimolecular Fluorescence Complementation (BiFC) assay, with each protein fused to a GFP half. Most of the pCX, pXC, pNX, and pXN construct combinations yielded a positive fluorescence signal by complementation. Fig. 4 shows those positive for both Arabidopsis and pea proteins, confirming also the absence of signal in the respective negative controls. The positive interaction was observed mainly in the nucleus, but was absent in the nucleole, and, particularly for pea proteins, speckles were detected in the nucleus in some of the combinations, such as *pNPsPYR1* + *pPsTRXo1C*, *pNPsTRXo1* + *pPsPYR1C*, and *pNPsTRXo1* + *pCPsPYR1*. Some signals were also present in the cytoplasm and vesicle-associated, such as *pNAtTRXo1* + *pCAtPYR1* or *pPsTRXo1N + pPsPYR1C* (detailed panels in Fig. 4).

### 3.2 Redox treatments of recombinant PYR1

**Fig. 5:**
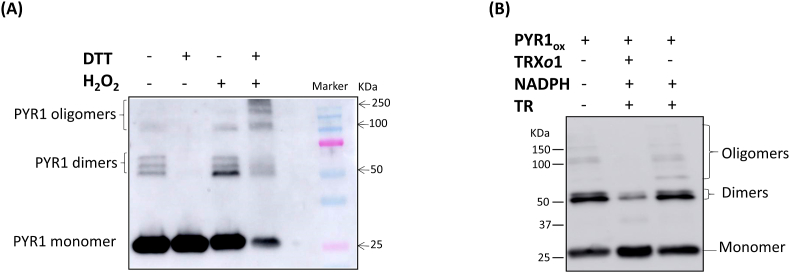
(A) Redox treatment of PYR1 and (B) reduction of PYR1 by the thioredoxin system. (A) Recombinant HisAtPYR1 (lane 1 without any treatment) was treated with 2 mM DTT (lane 2), or 4 mM H_2_O_2_ (lane 3), or firstly with 2 mM DTT before being treated with 4 mM H_2_O_2_ (lane 4), as described in Materials and methods. The pattern of PYR1 oligomerization after treatments is evidenced by the presence of the different monomeric, dimeric, and oligomeric forms, as pointed at the left side. Molecular weight markers are shown in the last lane and pointed on the right side. (B) Recombinant HisAtPYR1 was firstly reduced with 2 mM DTT before being oxidized with 4 mM H_2_O_2_ (PYR1_ox_) (lane 1) as described in Materials and methods. After this, PYR_ox_ was treated with TRX*o*1/NTR/NADPH (lane 2) or with NTR/NADPH (lane 3) as a control. The different oligomeric forms are pointed at the right side and the molecular weight markers on the left side.

Recombinant HisAtPYR1 protein was subjected to reducing or oxidizing treatments with DTT or H_2_O_2_, respectively, and the redox state was analysed after SDS-PAGE by Western blot using anti-PYR1. The recombinant protein without any treatment showed a strong signal of a monomer band around 25 kDa, dimeric forms around 50 kDa, and a faint signal around 100 kDa (pointed as oligomeric) (Fig. 5A first lane). When the protein was treated with 2 mM DTT (second lane), the dimeric and oligomeric forms decreased and were scarcely detected. H_2_O_2_-treated protein showed increased amounts of dimeric forms and decreased amounts of monomeric band. When the protein was previously reduced before being oxidized, the monomeric band highly decreased, whereas the oligomeric bands increased (fourth lane), appearing higher molecular weight bands. These results support the reversible redox modulation by DTT and H_2_O_2_ of the oligomeric state of PYR1 protein.

In order to study the capacity of the physiological NTR/TRX*o*1 system to reduce PYR1 instead of DTT, we analysed the oligomeric pattern of a previously oxidized recombinant HisAtPYR1 protein (PYR1_ox_) treated with the TRX system NADPH/TRX*o*1/NTRB. DTT-reduced PYR1 protein was oxidized with H_2_O_2_ treatment leading to similar amounts of the dimeric and monomeric forms of the protein, but low levels of oligomers were detected (lane 1 in Fig. 5B). When oxidized PYR1 was incubated with the TRX system, the oligomeric forms disappeared, the dimeric forms decreased, and the monomeric forms increased (lane 2 in Fig. 5B). Changes in the oligomeric, dimeric, and monomeric forms of the receptor were strictly dependent on the reduction catalysed by TRX*o*1, as no significant changes in the pattern of bands with regards to that of PYR1_ox_ were detected in the presence of NADPH and NTR but in the absence of TRX*o*1 (lane 3 in Fig. 5B).

**Fig. S4:**
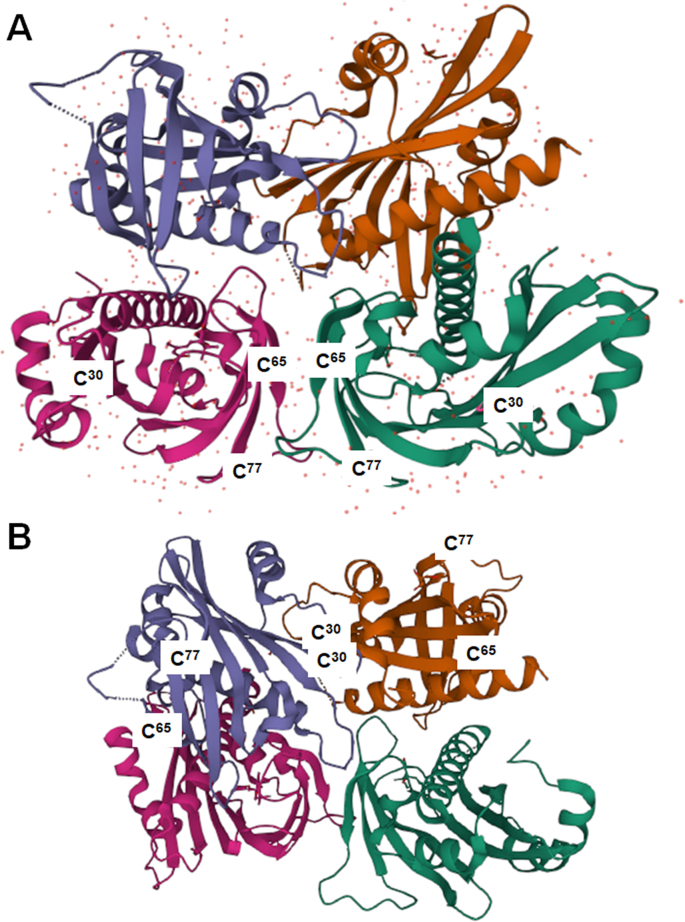
Model of the 3D structure of PYR1. The crystal structure of PYR1 in complex with ABA (experimental PDB structure 3K90) formed by two dimers organized in a tetrameric crystallographic unit. Only the magenta PYR1 monomer has an ABA molecule bound to the binding pocket. The position of cysteine residues in the different monomers is indicated. View showing the proximity of C^65^ residues (A) and C^30^ residues (B) in the different orientations of the monomers in the tetramer structure.

To analyse the presence of S–S bridges in the recombinant HisAtPYR1 protein, bottom-up mass spectrometry analysis was performed after DTT, H_2_O_2_, and dimedone plus H_2_O_2_ treatments. The disulfide containing dipeptides were quantified by counting their number of peptide spectral matches (PSMs). The non-treated protein presented a low number of PSMs between Cys^65^-Cys^7^^7^, whereas no S–S were detected in the DTT-treated protein (Table 1). In H_2_O_2_ and H_2_O_2_ + dimedone-treated samples, different S–S bridges were detected, with the highest number of PSMs being found between Cys^65^-Cys^65^, followed by Cys^30^-Cys^65^, while low amounts were detected for Cys^30^-Cys^30^ and Cys^65^-Cys^77^. These results point to a major disulfide bond formation between two Cys^65^ residues of different monomers linked to a homodimerization of the protein. Moreover, the existence of disulfide Cys^30^-Cys^65^ bonds is likely suggesting that oligomers are formed by the addition of new PYR1 monomers to already formed dimers by binding the free Cys^30^ residues of the dimers to Cys^65^ residues of new monomers, even though the identification of other disulfide bonds within other residues does not allow us to rule out other forms of oligomerization. Some of the possible forming S–S bridges are presented in Fig. S4 after modelling of a tetramer structure.

**Table 1:** Analysis of the presence of disulphide (S–S) bridges in the recombinant HisAtPYR1 protein subjected to reductive (DTT), oxidative (H_2_O_2_), and dimedone (DMD) + H_2_O_2_ treatments. ND, not detected.

	Cys^30^-Cys^30^	Cys^65^-Cys^65^	Cys^30^-Cys^65^	Cys^65^-Cys^77^
	(PSMs)			
PYR1	ND	ND	ND	Low number
PYR1 + DTT	ND	ND	ND	ND
PYR1 + H_2_O_2_	4	89	13	1
PYR1 + DMD + H_2_O_2_	4	83	17	2

In order to check the involvement of each of the three cysteine residues in the oligomerization of PYR1, we produced the recombinant mutated proteins HisAtPYR1C30S (C30S), HisAtPYR1C65S (C65S), and HisAtPYR1C77S (C77S), in which cysteine was replaced by serine, and the proteins were subjected to the same reducing (DTT) and oxidizing (H_2_O_2_) treatments that were described above.

**Fig. 6:**
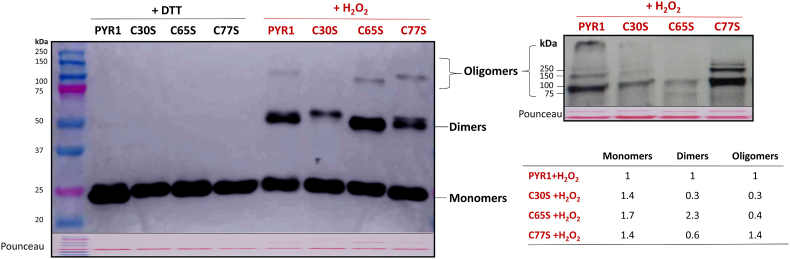
Redox treatment of recombinant mutant variants of HisAtPYR1 in which one cysteine has been replaced by serine. Recombinant wild type rAtPYR1 and mutated AtPYR1C30S (C30S), HisAtPYR1C65S (C65S), and HisAtPYR1C77S (C77S) were treated with 2 mM DTT (first 4 lanes in gel on the left) or 2 mM DTT + 4 mM H_2_O_2_ (lanes 5–8) as described in Materials and methods. Oligomeric and monomeric forms are pointed, as well as molecular weight markers. Pounceau staining is shown to check loading. Gel on the right is an example with an excess of protein for a better visualization of the oligomers, and table presents the relative quantification of the bands in three independent experiments of the H_2_O_2_-treated proteins.

All of the recombinant proteins presented an increase in oligomeric bands (first four samples compared to the last four in gel on the left in Fig. 6) when subjected to an oxidative treatment as previously shown for wild type recombinant PYR1 protein (Fig. 5). All of the mutated proteins presented higher amounts of monomer than WT PYR1, but differed in the dimeric forms: while C30S and C77S displayed less dimers (0.3- and 0.6-fold, respectively) than PYR1, C65S had the highest amount of dimers (2.3-fold compared to PYR1). All of them presented oligomeric forms in lower abundance, although with evident differences (left panel, Fig. 6), so we decided to load higher amounts of proteins for a better visualization of oligomers. A high molecular mass oligomer of more than 250 kDa was detected in PYR1 and, to a lesser extent, in C30S, but it was not present in C65S or C77S (right panel, Fig. 6). Oligomers for C77S protein were similar to that of PYR1 protein, yet with different intensity pattern. In turn, C30S and C65S proteins formed much less oligomers than PYR1 (right panel, Fig. 6). These results suggest that the oligomerization process of PYR1 in response to oxidative conditions happens mainly through the formation of intermolecular disulfide bonds between C^30^ and C^65^ residues of different receptor molecules.

### 3.3 Modulation of PYR1 activity by TRXo1

**Fig. 7:**
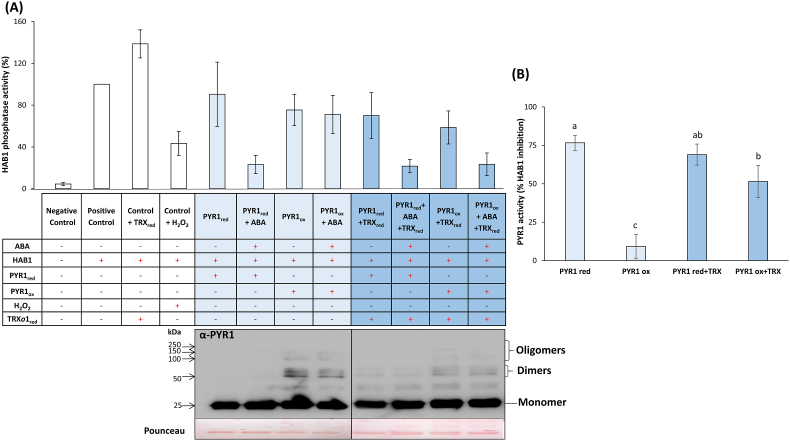
(A) HAB1 phosphatase and (B) PYR1 activity in the presence/absence of recombinant AtPYR1, PsTRX*o*1 or ABA, under reducing (red) or oxidizing (ox) conditions. Values in A represent the phosphatase activity percentage in the reaction using different components specified in each case, in comparison with the HAB1 phosphatase activity alone (positive control considered as 100%). PYR1 and TRX_o_1 proteins were reduced with 2 mM DTT when indicated (PYR1_red_ and TRX*o*1_red_) and PYR1 was previously reduced with 2 mM DTT and, after desalting, it was oxidized with 4 mM H_2_O_2_ (PYR1_ox_) as described in Materials and methods. HAB1 phosphatase activity was measured using pNPP as substrate monitoring changes in absorbance at 405 nm. Western blot against anti-PYR1 antibody shows an example of the oligomeric forms detected in the different reactions tested for the measurement of the HAB1 phosphatase activity. Pounceau staining is shown to check loading. Values in B represent the PYR1 activity measured indirectly through the inhibition of HAB1 phosphatase activity. The percentage of PYR activity was calculated by comparing the PYR1 activity in the presence and absence of ABA for each reaction (from data in Fig. 7A). Values are the mean ± SE of 3 replicates. Different letters indicate significant differences (p < 0.05) among treatments according to the Tukey's test. (For interpretation of the references to colour in this figure legend, the reader is referred to the Web version of this article.)

To check whether PYR1-TRX*o*1 interaction led to an effect on the activity of PYR1 as ABA receptor and PP2C inhibitor, we analysed its activity based on coupled assays with HAB1 phosphatase. PYR1 displays inhibitory activity on PP2C phosphatases when it is bound to the phytohormone. The activity of this receptor was then measured *in vitro* by a coupled indirect assay analysing the activity of HAB1 phosphatase after incubation with recombinant AtPYR1 previously reduced with DTT (red) or oxidized with H_2_O_2_ (ox). The positive control corresponding to HAB1 was considered as 100% of its activity (Fig. 7A). When the reaction was done in the presence of recombinant PsTRX*o*1 previously reduced by DTT (Control + TRX_red_) an activation was observed, probably due to the reduction of the oxidized fraction of HAB1, as H_2_O_2_-triggered oxidation inhibited HAB1 activity (Fig. 7A). Then we analysed whether the redox state of PYR1 influenced its inhibitory effect on HAB1 activity. For each reaction, an internal control was carried out in the absence of ABA and the values were used for calculation of the PYR1 activity. When PYR1 was reduced and in the presence (+) of ABA, HAB1 activity was inhibited, presenting around 20% of the activity related to the positive control (100%), showing that the PYR1 was active (Fig. 7B). However, when PYR1 was oxidized (PYR1_ox_), the activity was around 75% of that of the positive control (Fig. 7A) implicating that PYR1_ox_ activity was lower than PYR1_red_ (Fig. 7B). To check whether a physiological reductant as TRX*o*1 can restore the activity of oxidized PYR1 as DTT did, we conducted the assay in the presence of PsTRX*o*1 recombinant protein previously reduced by DTT. The addition of DTT-reduced PsTRX*o*1 with DTT-reduced PYR1 and ABA did not affect the inhibitory effect on the phosphatase activity (Fig. 7A). However, when reduced TRX*o*1 was incubated with less active PYR1_ox_, a recovery of the receptor-triggered inhibition of HAB1 was observed, reaching similar values to those detected after chemical reduction of PYR1_ox_ with DTT (Fig. 7A and B). The oligomerization pattern of the receptor in the different phosphatase assay reactions shown above were checked by Western blot using anti-PYR1 antibody. The presence of dimeric forms of PYR1 in oxidized samples was partially removed after incubation with TRX*o*1, likely due to the TRX*o*1-mediated reduction and the consequent reversion of dimers to monomers (Fig. 7A). These results revealed that both HAB1 and PYR1 functions were redox-regulated by TRX*o*1. Therefore, the interaction TRX*o*1-PYR1 could have a physiological role in the modulation of PYR1 activity and, consequently, ABA signalling.

### 3.4 In vivo analysis of PYR1 oligomeric state

**Fig. 8:**
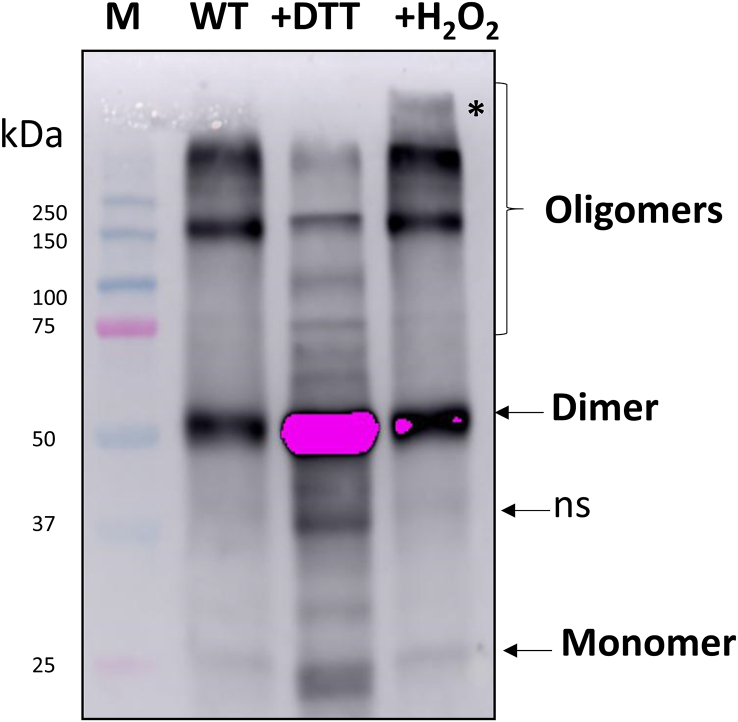
Western blot analysis for *in vivo* PYR1 detection. Western blot was performed using α-PYR1 antibody in WT plants in non reducing, reducing (+DTT), and oxidizing (+H_2_O_2_) treatments of the leaf extracts. Extracts (lane 2) were incubated with 50 mM DTT (lane 3) or 4 mM H_2_O_2_ (lane 4). Marker (M) proteins were loaded in lane 1 for molecular weight position. The pattern of PYR1 oligomerization after treatments of the leaf extracts is evidenced by the presence of the different monomeric, dimeric, and oligomeric forms, as pointed at the right side. The asterisk points a very high molecular weight oligomer and ns points a non-specific band.

We examined the oligomeric state of PYR1 in wild type Arabidopsis with non-reducing SDS-PAGE. PYR1 was detected in a leaf extract without using any blocking or alkylating agent, mostly as a dimer form of around 50 kDa and much less as a monomer of 25 kDa and several bands of oligomers (Fig. 8). When 50 mM DTT was added to the extract, a stronger signal in the dimeric and monomeric forms was observed (lane 3), together with several intermediate bands, probably due to the reduction of disulfide bridges from the oligomeric forms. When 4 mM H_2_O_2_ was added to extracts, a decrease in the PYR1 monomer and an increase in high molecular mass oligomeric complexes were detected with regards to the DTT-treated sample (Fig. 8). These data support that *in vivo* redox changes of PYR1 protein are reversible.

**Fig. 9:**
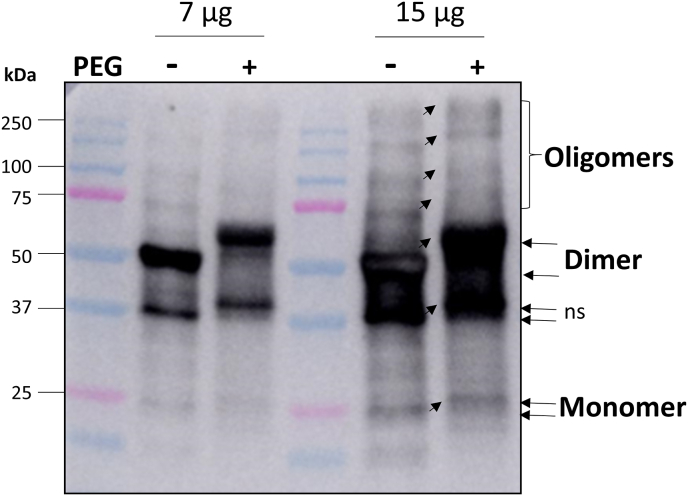
Western blot analysis for *in vivo* PYR1 detection in WT leaf extracts after a redox shift assay. Western blot was performed using α-PYR1 antibody in extracts treated with NEM (blocking agent of free cysteine thiols) + TCEP (as reductant of S–S groups) in the absence and presence of MM[PEG]24 (alkylating agent adding molecular weight to free cysteine thiols). Two different amounts of protein are presented for a better visualization of the different bands, and the monomer, dimer, and oligomer forms of PYR1 are pointed together with a non-specific (ns) band. Marker (M) proteins were loaded in lanes 1 and 4 for molecular weight position.

To analyse the presence of a disulfide bridge in the protein *in vivo*, we performed redox shift mobility assays on protein extracts from WT Arabidopsis plants using NEM to block the reduced thiols and TCEP to reduce the possible disulfide bridge, labelling the resulting thiols with MMPEG. As a result of the treatments, PYR1 monomer band shifted to a molecular weight around 2 kDa higher, the dimers also changed the mobility around 6 kDa higher, and even the oligomers appeared to have a higher mobility than the protein without the alkylating treatments (Fig. 9).

Changes in the mobility of all of the detected bands indicated the existence of at least a disulfide bridge in the proteins. Regarding the monomer, an intramolecular bridge was detected, likely between Cys^65^ and Cys^77^, which are the closest Cys residues in the 3D structure (Fig. S4).

### 3.5 Characterization of transgenic Arabidopsis plants overexpressing AtTRXo1

**Fig. 10:**
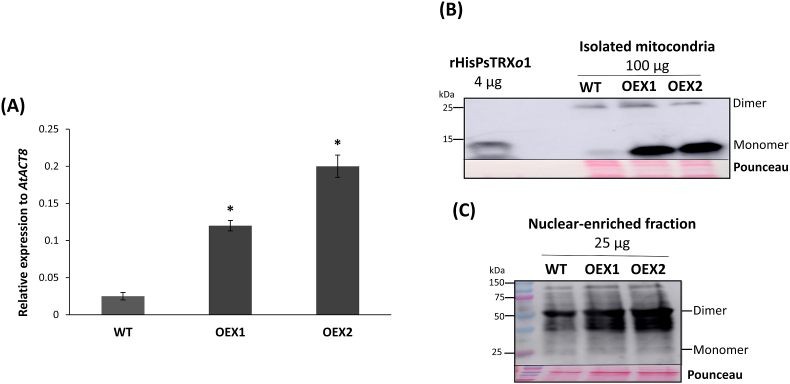
Analysis of over-expression of *AtTRXo1.* (A) Gene expression analysis by RT-qPCR of *AtTRXo1* relative to the *ACTINE 8* (*AtACT8*) gene. Expression data are the mean ± SE of three technical replicates of five biological samples (with a pool of 10 plants in each sample). Asterisk indicates that data significantly different according to t-Student's test (P < 0.05). Western blot analysis of mitochondrial (B) and nuclear-enriched (C) proteins from wild type (WT) and over-expressing (OEX) *Attrxo1* lines using the AtTRX*o*1 antibody. Recombinant (r) pea PsHisTRX*o*1 protein was loaded as a control in B and Pounceau staining of the membranes is shown as a loading control. Marker (M) proteins were loaded and marked in each membrane for molecular weight position.

Trying to decipher the biological function of TRX*o*1 with regards to ABA signalling, afterwards we decided to examine the response to ABA of two KO (SALK lines) and overexpressing (OEX, obtained in our laboratory) *AtTRXo1* mutants. To check over-expression, we performed an analysis of gene expression by RT-qPCR as well as Western blot analysis of isolated mitochondria and nuclear-enriched fractions from Arabidopsis leaves. As shown in Fig. 10A, both transgenic OEX lines presented a high level of *AtTRXo1* transcript compared to WT plants. At the protein level, OEX lines showed a higher amount of mitochondrial (mainly monomers around 13.5 kDa as reported for the mature protein [41] (Fig. 10B) and nuclear (monomer around 25 kDa and mainly dimers around 50 kDa) (Fig. 10C) TRX*o*1 protein. These results allowed us to validate the over-expression at mRNA and protein levels in the two OEX lines.

### 3.6 Oligomerization pattern of PYR1 in response to ABA

**Fig. 11:**
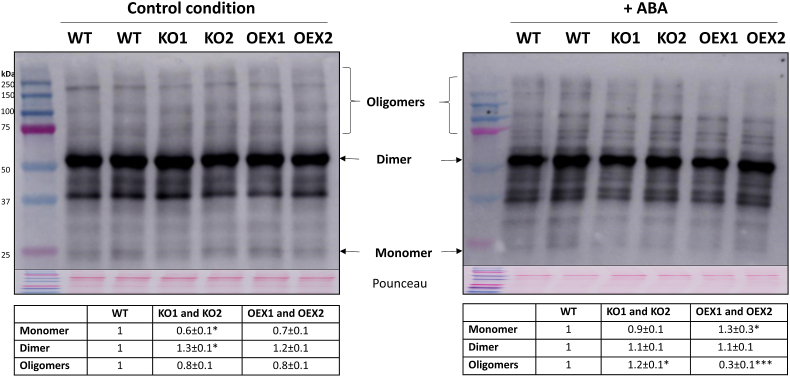
Western blot analysis for *in vivo* PYR1 detection in leaf extracts. Western blot was performed using α-PYR1 antibody in leaf extracts of WT, 2 lines of KO and 2 lines of OEX *Attrxo1* mutant plants grown in plates in the absence and presence of 10 μM ABA (+ABA) for 48 h. Marker (M) proteins were loaded in lane 1 for molecular weight position and Pounceau staining is shown to check loading. The pattern of PYR1 oligomerization of the leaf extracts is evidenced by the presence of the different monomeric, dimeric, and oligomeric forms, as pointed. Tables under the membranes present the band quantification as a mean of three independent experiments ± SE, and asterisks represent significant differences with WT (value 1) using t-Student's test (P < 0.05 [*], P < 0.005 [***]). The bands higher than 75 kDa are considered as oligomers for quantification.

**Fig. S5:**
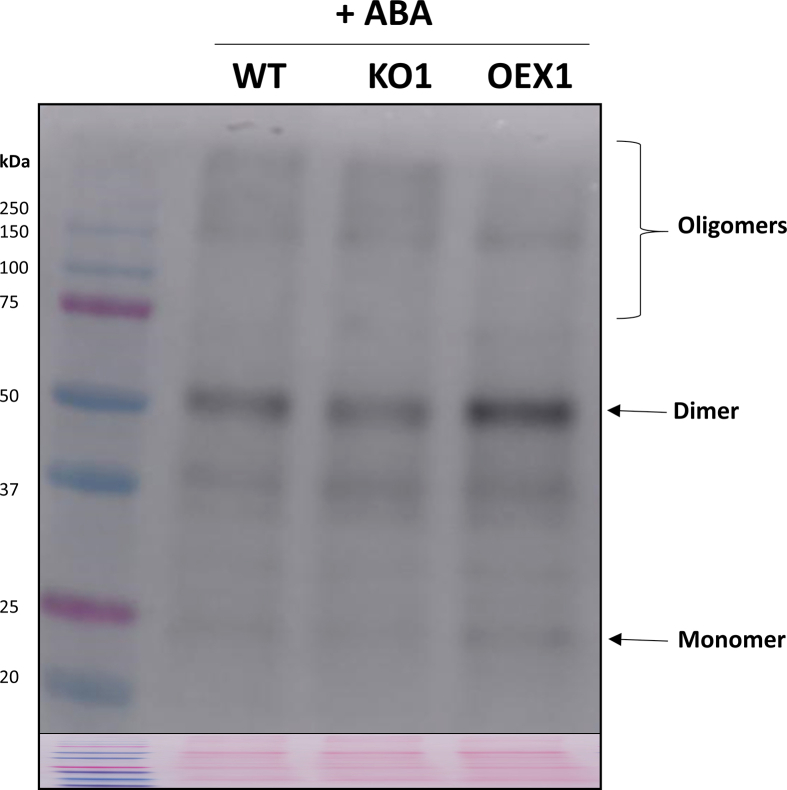
Western blot analysis for PYR1 detection in leaf extracts. Western blot was performed using α-PYR1 antibody in leaf extracts of WT, KO1, and OEX1 *Attrxo1* mutant plants grown in the presence of 10 μM ABA (+ABA) for 48 h. Marker (M) proteins were loaded in lane 1 for molecular weight position, and Pounceau staining is shown to check loading.

To explore the possible physiological implication of TRX*o*1 in the oligomerization pattern of PYR1 *in vivo* in response to ABA, we analysed the oligomeric state of PYR1 in the two KO and OEX *Attrxo1* mutant lines compared to the WT genotype, in leaf extracts of the plants after 48 h growing in the presence of 10 μM ABA (+ABA, Fig. 11). A control experiment was also carried out in all of the genotypes growing in the absence of ABA (control conditions, Fig. 11). No apparent differences were visualized in control conditions among genotypes in the different oligomerization forms of the PYR1 protein (Fig. 11, membrane on the left), although quantification of the bands of three independent experiments revealed that KO *Attrxo1* mutants presented a lower amount of monomer and a higher amount of dimer than the WT genotype (table under membrane on the left in Fig. 11). When plants were grown in the presence of ABA for 48 h (Fig. 11, membrane on the right), KO plants had a significantly higher amount of oligomers (≥75 kDa), whereas OEX plants presented a visibly lower signal of these forms of the protein than WT plants, more evident in those of a higher molecular weight (more than 250 kDa). This was confirmed by the quantification values (table under membrane on the right in Fig. 11). The dimeric form was similar in all of the genotypes, whereas the monomeric form was slightly higher in the OEX plants. Due to the high amount and signal of the dimeric form of the protein, we performed Western blots loading lower amount of protein to better visualize the signal (an example is shown in Fig. S5).

## 4 Discussion

In this work, affinity chromatography, *in vitro* Dot-blot, and co-immunoprecipitation assays, as well as *in vivo* BiFC experiments, have demonstrated that Arabidopsis and pea TRX*o*1 interact with PYR1 in the cell nucleus. Although the TRX system was described in chloroplast and most of the work on these proteins has been focused on their role in this organelle, in recent decades, the involvement of TRXs has been extended towards its function in the mitochondria, cytoplasm and, to a lesser extent, the nucleus [[42], [43], [44], [45]]. Specifically, in the nucleus, we previously reported the presence of TRX*o*1 in pea plants and TBY-2 cell suspension culture together with a localization in mitochondria [25,26], while in Arabidopsis plants, AtTRX*o*1 was reported only in mitochondria [41]. In this context, the interaction shown in the nucleus of AtTRX*o*1 and AtPYR1 is an indication of the dual location of this type of TRX*o*1 also in Arabidopsis. In fact, our study in isolated mitochondria and enriched-nuclear fractions from leaves of OEX *Attrxo1* mutants revealed the presence of TRX*o*1 in these cellular compartments, being the mitochondrial isoform shorter (around 13.5 kDa as previously described [41], whereas the nuclear form presented a higher molecular weight (around 25 kDa), corresponding to the previously described mature form of the protein (including the mitochondrial signal peptide that is not processed), although, as described above, this form was not specifically localized in the nucleus previously. Data related to the function of TRX*o*1 in this organelle are very scarce. We recently reported PCNA (proliferating cell nuclear antigen) as a new target of this TRX*o*1 protein [26], extending its function to DNA replication and cell proliferation. Aside from this evidence, the presence of other redox systems in the nucleus has been shown for 1-Cys PRX and NTRA [46,47], together with TRX*h* and NTR in wheat aleurone and esculent cells in a situation of oxidative stress such as that associated with germination, moving from the cytosol to the nucleus [48,49]. Also a TRX*h*5 localized in both the cytoplasm and the nucleus in Arabidopsis subjected to moderate oxidative stress was in agreement with the movement of its nucleocytoplasmic substrate NPR1 regulating its oligomerization state [50]. In this scenario, and as we have commented, the scope of the function of TRX*o*1 in the nucleus is barely known, so the results that we have obtained verifying the interaction with PYR1, the ABA receptor, as well as the effect that this interaction has on the activity of this sensor and its oligomerization state, represent an important advance in the study of the ABA signalling pathway and the role of TRX in the nucleus, taking into account that the redox regulation of ABA receptors has not been described.

Different evidences point to the oligomerization state of ABA receptors as a key event in their function. PYR1 together with PYL1 and PYL2 have been reported as dimeric proteins, while other PYL members are monomeric. In the absence of ABA, recombinant PYR1 forms homodimers in solution, even if crystallization of the protein was successful only in the presence of ABA, forming an asymmetric unit of one ABA-bound and one ABA-free subunits [51]. Even for the dimeric forms of the receptors, crystallographic studies have shown a receptor–ABA–phosphatase complexes 1:1:1 stoichiometry, which implies a step of dissociation of the dimer necessary for receptor activation and signalling [51,52]. After transient transformation of *Nicotiana benthamiana* plants, these authors reported that PYR1 formed a dimer *in vivo*, both in the presence and in the absence of applied ABA. Moreover, in spite of the described low basal activity of dimeric receptors to elicit PP2C inhibition in comparison to monomeric ones, a quadruple mutant *pyr1/pyl1/pyl2/pyl4* showed compromised aspects of ABA signalling, evidencing the importance of dimeric receptors in ABA-related processes [7]. In fact, more recently, a deeper study of the differences between monomeric and dimeric receptors has shown that in the dimeric forms, ABA acts as an allosteric promoter for the needed gate closure, which leads to dissociation of the two subunits allowing interaction and inhibition of PP2Cs [53]. In our plants, the fact that PYR1 is present mainly as a dimeric form, with less abundance in monomeric and oligomeric forms, is in accordance with the proposed key function of dimerization in receptors like PYR1 and PYL1 to prevent basal interactions with PP2Cs in the absence of ABA [54]. Interestingly, these last authors performed the experiments in the presence of reductants such as β-mercaptoethanol, an aspect that may imply a possible modulation of the thiol state of the receptor. In fact, Arabidopsis PYR1 contains three Cys residues in its amino acid sequence, although its redox modulation has not been described. In this work, we have demonstrated by Western blot analysis that recombinant PYR1 is redox-regulated by H_2_O_2_ and reductants such as DTT, but, interestingly, also by the NTR/TRX*o*1 system *in vitro*, and the Cys residues involved in its oligomerization have been also identified. Moreover, oligomeric state is regulated by oxidation-reduction and the activity of the receptor on HAB1 phosphatase in the presence of ABA is also modulated by TRX*o*1, implying a new role for redox regulation and for *in vivo* reductants such as the TRX system including TRX*o*1 in PYR1-mediated ABA perception. Also of interest is the fact that *in vivo* PYR1 protein showed a different oligomeric pattern when leaf extracts were subjected to reductive and oxidative treatments. Accordingly, the fact that OEX *Attrxo1* plants presented less oligomers and more monomers when growing in the presence of ABA indicated that TRX*o*1 has a role in the oligomeric state of the receptor, facilitating the conformation of a functional PYR1 protein with a possible positive consequence on ABA-related processes. Interestingly, dimers were predominant in all of the genotypes, so the reduction of oligomeric forms to dimers seems to be more important than that from dimers to monomers, in accordance with the description about the stabilization of the dimers as a key event in the *in vivo* function of the receptor. Related to this, it has been shown that apo-PYL3 and (+)-ABA-bound-PYL3 are mainly in dimer conformation in solution, and that monomer conformation is necessary for the inhibitory effect of the receptor on HAB1 [13]. More recent computational simulations have revealed that, since the dimerization surface is also involved in binding to PP2Cs, it is impossible for them to interact with dimeric receptors in a direct way, so the interaction requires the dimer to dissociate [53]. Regarding the role of TRX in the nucleus and in the structure of oligomers, recently TRX*h*2 has been described to translocate to this organelle under cold stress to interact with all of the oxidized inactive forms of CBFs (C-repeat binding factors), including oligomers and monomers. The active reduced monomers are then able to activate cold-regulated gene expression [55] conferring tolerance. Considering the refined strategy of plants against cold snap, it is interesting to extend to other stress situations the described idea that CBFs oligomers present in control conditions could be considered as a repository that exists in plants to protect them from sudden temperature drops. In this sense, other redox post-translational modifications on specific amino acid residues in target proteins apart from Cys oxidation, such as S-nitrosylation, S-glutahionylation, persulfidation or Tyr nitration, are emerging as key regulators of signal transduction, allowing cell response under an imbalance of redox homeostasis. In this way, regulated proteins involved in different metabolic pathways are able to sense and transmit the imbalance occurring in response to biotic and abiotic stresses [15,21,[56], [57], [58]]. Related to ROS-induced modifications, a mild oxidative stress causes the oxidation of Cys residues in proteins to generate sulfenic forms or disulfide bridges, both reduced to –SH by different cellular reductants. Given the eminently reducing nature of TRX*o*1 and its verified interaction with PYR1 in the nucleus, the study of the redox regulation of this receptor led us to verify that oxidation strongly reduced its activity and that TRX*o*1 was able to reactivate it through reduction. Thus, redox-related processes on PYR1 constitute a novel post-translational regulation for this receptor. Although the information concerning redox regulation of PYR1 is rather scanty, it was previously reported that nitration of key tyrosine residues of PYR1, as well as other receptors of the family, rapidly inactivates the receptor function and promotes their polyubiquitination and further proteasomal degradation [15,[59], [60], [61]]. With regards to S-nitrosylation, it did not affect the inhibition of PP2C by the receptor, and ABA signalling was not inhibited [15]. To our knowledge, redox modulation has not been reported for ABA perception. Moreover, PYR1 was reported by Waszczak et al. [62] among the sulfenilated proteins in the sulfenome of Arabidopsis cell suspension culture and by Aroca et al. [19] as persulfidated in Arabidopsis WT leaf extracts under basal conditions, although the effect of these PTMs has not been analysed. In fact, a connection between H_2_S and ABA has been demonstrated in plants under stress conditions in which protein persulfidation is triggered, and proteomic studies have revealed that the persulfidation of several proteins in Arabidopsis is involved in ABA signalling, including PYR1, PYL1 as well as SnRK2.2 and OST1 protein kinases and HAB2 and ABI4 phosphatases [19, 20, 63].

As a summary of our results, the demonstrated formation of oligomeric forms of PYR1 by oxidation with the consequent loss of activity on HAB1 and recovery by reduction, the identification of the Cys involved in dimerization and oligomerization of the receptor, the *in vivo* formation of oligomers after H_2_O_2_ treatment of leaf extracts, the demonstrated interaction of PYR1 with TRX*o*1 in the nucleus, and the involvement of TRX*o*1 in the reduction of oligomers *in vitro* and *in vivo* in OEX *Attrxo1* plants, all point to a novel redox regulation of ABA receptors as PYR1 favouring ABA signalling. As we previously commented, these results are really ground-breaking due to the unknown regulation of this PTM on ABA sensors, being TRX*o*1 a good candidate as the physiological reductant *in vivo* of these receptors in the nucleus.

## 5 Conclusions

Results presented in this work imply that redox regulation may be a key event modulating the function of ABA receptors such as PYR1. At least *in vitro*, TRX*o*1 is able to rescue the activity lost by oxidation of this sensor and, thus, the interaction TRX*o*1-PYR1 may have a physiological function in the redox modulation of the ABA signalling. The oligomeric structure of the receptor is modulated by the TRX system in order to exert its function on PP2Cs to allow the cellular ABA response, being mainly two of the three Cys of PYR1 involved in its dimerization and oligomerization. These results point to the existence of a new redox regulation on the signalling pathways triggered by ABA, which may affect processes as important as seed germination, plant growth and stomatal behaviour, determinant for plant response to stress situations. Research in this line will favour the advance in the knowledge of ABA signalling routes in order to improve the response of plants to increasing aggressive adverse situations faced nowadays by plant production, such as water deficit or salinity.

## Author contributions

A.J., F.S., and J.L. designed the research; S. D-B, A. S-G., M-C. C., and D. V. performed the experiments; S. D-B, A. S-G., M-C. C., F.S., and A.J. analysed the data; A.J., F.S and J.L. wrote and revised the manuscript.

## Funding sources

This study was supported by the Spanish grants MICINN-FEDER (PID2021-127335NB-I00, PID2020-112618 GB-I00 (to JL) and Network RED2018-102397-T) and Séneca Foundation (Excellence Project 19876/GERM/15 and project 22051/PI/22).

## Declaration of competing interest

None.

## Data Availability

Data will be made available on request.
