# Supplementary material for: Thioredoxin TRXo1 is involved in ABA perception via PYR1 redox regulation
Source: Redox Biol. 2023 May 26;63:102750. doi: 10.1016/j.redox.2023.102750 (PMC10244696; doi:10.1016/j.redox.2023.102750)
Supplement: Multimedia component 1 [file mmc1.docx]

**Table S1.** Oligonucleotides used in cloning and RT-qPCR analysis.

| **ID No.** | **Name** | **Sequence 5’-3’** |
| --- | --- | --- |
| 1 | *Qt-adapter* | CCAGTGAGCAGAGTGACGAGGACTCGAGCTCAAGC(T)17 |
| 2 | *Q_0_-tailS1* | CCAGTGAGCAGAGTGACG |
| 3 | *Q_1_-tailS2* | CAGGACTCGAGCTCAAGC |
| 4 | *Q_0_-GSP1* | CGCACACAACACCACCTAAC |
| 5 | *Q_1_-NGSP1* | TGAAAGTCGGTTGCACCAGA |
| 6 | *Q_1_-NNGSP1* | CAATGTCATCTCCGGTCTCC |
| 7 | *RT-GSP 5`RACE* | CATCAACTATATAAGAT |
| 8 | *UAP-adapter* | GGCCACGCGTCGACTAGTACCGGG55GGG55GGG55G |
| 9 | *Q_0_-GSP2* | GCTTCACAACGGTATCAGCA |
| 10 | *Q_1_-NGSP2* | GCAAAAAGACGCGTATCCTC |
| 11 | *Q_1_-NNGSP2* | CAGAACAACCGTCCGGATCT |
| 12 | *AtPYR1-ORF-*  *XbaIFw* | AGTCTAGAATGCCTTCGGAGTTAACACC |
| 13 | *AtPYR1-ORF-*  *BamH1Rv* | TCGGATCCTCACGTCACCTGAGAACCAC |
| 14 | *PsTRXo1_ORF_*  *XbaIFw* | AGTCTAGAATGGTTGGAACCAGAAATTTG |
| 15 | *PsTRXo1_ORF_*  *BamH1Rv* | TCGGATCCTTAGTCCTTCTTGAAGAGTT |
| 16 | *AtTRXo1_ORF_*  *XbaIFw* | AGTCTAGAATGAAGGGAAATTGGTCGAT |
| 17 | *AtTRXo1_ORF_*  *BamH1Rv* | TCGGATCCTCACTTGTAGAGCTGTTCCA |
| 18 | *AtTRXo1non-STOPBamH1Rv* | TCGGATCCCTTGTAGAGCTGTTCCATGA |
| 19 | *PsTRXo1non-STOPBamH1Rv* | TCGGATCCGTCCTTCTTGAAGAGTTTCT |
| 20 | *AtPYR1non-STOPBamH1Rv* | TCGGATCCCGTCACCTGAGAACCACTT |
| 21 | *PsPYR1_ORF_XbaIFw* | AGTCTAGAATGGAGAAAGCAGAGAGCT |
| 22 | *PsPYR1non-STOPBamH1Rv* | TCGGATCCATGTGACTTACCGTCACCGT |
| 23 | *PsPYR1_ORF_BamH1Rv* | TCGGATCCCTAATGTGACTTACCGTCAC |
| 24 | *AtTRXo1gw_ORF_Fw* | AAAAAAGCAGGCTTCATGAAGGGAAATTGGTCGAT |
| 25 | *PsTRXo1gw_ORF_Fw* | AAAAAAGCAGGCTTCATGGTTGGAACCAGAAATTTG |
| 26 | *AtPYR1gw_ ORF_Fw* | AAAAAAGCAGGCTTCATGCCTTCGGAGTTAACACC |
| 27 | *PsPYR1gw_ORF_Fw* | AAAAAAGCAGGCTTCATGGAGAAAGCAGAGAGCT |
| 28 | *AtTRXo1nSTPgw_Rv* | CAAGAAAGCTGGGTCCTTGTAGAGCTGTTCCATGA |
| 29 | *PsTRXo1nSTPgw_Rv* | CAAGAAAGCTGGGTCGTCCTTCTTGAAGAGTTTCT |
| 30 | *AtPYR1nSTPgw_Rv* | CAAGAAAGCTGGGTCCGTCACCTGAGAACCACTT |
| 31 | *PsPYR1nSTPgw_Rv* | CAAGAAAGCTGGGTCATGTGACTTACCGTCACCGT |
| 32 | *PYR1-C30S-Fd* | CAGGAAGCTCATCATCACTC |
| 33 | *PYR1-C30S-Rv* | GAGTGATGATGAGCTTCCTG |
| 34 | *PYR1-C65S-Fd* | TCAAATCCTCGTCCGTCGAA |
| 35 | *PYR1-C65S-Rv* | TTCGACGGACGAGGATTTGA |
| 36 | *PYR1-C77S-Fd* | CGTCGGATCGACGCGC |
| 37 | *PYR1-C77S-Rv* | GCGCGTCGATCCGACG |
| 38 | *PYR1-NcoI-Fd* | aaAACCATGGCTTCGGAGTTAAC |
| 39 | PYR1-EcoRI-*Rv* | aaAAGAATTCTCACGTCACCTGAGAAC |
| 40 | *AtTRXo1for* | AAAAAAGCAGGCTTCATGAAGGGAAATTGGTCGATC |
| 41 | *AtTRXo1rev* | CAAGAAAGCTGGGTCCTATCACTTGTAGAGCTGTTCCAT |
| 42 | *AttB1tail5´* | GGGGACAAGTTTGTACAAAAAAGCAGGCTTC |
| 43 | *AttB2tail3´* | GGGGACCACTTTGTACAAGAAAGCTGGGTC |
| 44 | *AtTRXo1for* | TCGAAGAAAGGGGAGGTTG |
| 45 | *AtTRXo1rev* | CACTTGTAGAGCTGTTCCATGAG |
| 46 | *AtActina8for* | TGCCTGGACCTGCTTCATC |
| 47 | *AtActina8rev* | AGCTGCAGGGATCCACGAGA |
